# Healthcare Professionals' Culture Toward Reporting Errors in the Oncology Setting

**DOI:** 10.7759/cureus.38279

**Published:** 2023-04-29

**Authors:** Abdullah Bany Hamdan, Sherwynn Javison, Musa Alharbi

**Affiliations:** 1 Comprehensive Cancer Center, King Fahad Medical City, Riyadh, SAU

**Keywords:** professionals, oncology, culture, errors, safety, reporting

## Abstract

Introduction: Reporting an error during a hospital journey is crucial to reduce such errors' recurrence and to learn from events. Therefore, this study aimed to investigate oncology staff's attitudes, perceived barriers, and strategy towards reporting incidents and errors.

Methods: A cross-sectional online survey was conducted among health professionals providing care to cancer patients in a tertiary healthcare hospital in Saudi Arabia in 2019. Data were collected using an online self-administered questionnaire distributed to the targeted population.

Results: A total of 211 participated in this study. Sixty-five percent of responders reported that they felt a need to reveal errors. The leading perceived barrier to reporting the events was that the staff wanted to avoid getting into trouble (60%), followed by worries about legal action (59.2%). The top-ranking strategy to improve reporting by nurses was to have clear guidelines to report errors, education and feedback by doctors, and further education and training by allied healthcare.

Conclusion: The study revealed that healthcare professionals do possess a favorable attitude toward reporting errors. However, a major gap is still a barrier between attitude and practice, and this need creating a safe atmosphere where every healthcare professional feels safe and comfortable with reporting incidents is required to build a non-punitive environment to enhance the safety culture. On the other hand, the respondents listed different strategies to enhance reporting events and errors.

## Introduction

The oncology setting is complex and considered one of the high-risk areas with an increased chance of error due to the complexity of the therapeutic modalities and different processes [1]. Cancer patients and their caregivers are considered vulnerable as they are already in distress caused by the diagnosis [2]. Errors can occur anytime during the cancer patient's journey [3]. Voluntary reporting of medical errors by healthcare providers is an important strategy to enhance patient safety [4]. Nevertheless, few studies in the Middle East tackle healthcare provider attitudes, perceiving barriers to reporting incidence and errors in oncology settings. 

Medical errors are considered the fifth leading cause of death in the U.S. [5], and the errors arise from gaps and problems within the system and human errors that pose potential safety risks [6]. Also, medical errors are responsible for up to 251,000 deaths annually, accounting for 9.5% of all deaths in the U.S. [7]. Healthcare institutions are developing strategies to combat all forms of error, but despite different efforts to eradicate them, they still occur within healthcare settings and affect patients and other personnel [8]. 

A practical approach to reporting errors, incidence, and consequences of errors is crucial to enhancing patient safety culture in organizations [9-11]. Moreover, focusing on the successes and failures of the process rather than blaming others [12]. Additionally, these reports will be helpful to learn from events or errors as a lesson learned [13,14] to find the correct cause and improve safety practices [9]. 

In Saudi Arabia, there is an increasing incidence rate of medical errors, and medical liability claims [15]. The error can be classified into risky behavior, near misses, minor injury, severe injury, or fatality within a healthcare facility [16]. Furthermore, the effects of errors can vary from non to temporary discomfort, permanent disability, or death, based on the intensity and proximity of the error to the patient [17]. 

However, a study showed that 50% to 96% of medical errors are not reported, and 96% are not evaluated [18]. Several studies have shown that certain factors can act as barriers to reporting medical errors, such as decreased motivation or willingness, unclear unit values, lack of systematic analysis of mistakes, work overload, a lack of teamwork [19], fear of blame [20], and disciplinary actions [21]. 

Healthcare institutions should create a safe atmosphere where every healthcare professional can feel comfortable voicing their concerns [16]. Incident reporting does not limit the work environment for those who make a report or commit an error. Instead, it is an opportunity to gather information on clinical incidents to ensure that similar incidents do not occur again [8]. At the same time, voluntary reporting is a process of reporting safety events by anyone, being directly or indirectly involved, in a confidential manner [22]. 

In Saudi Arabia, the Ministry of Health and Patient Safety Center designed a set of strategies and processes to encourage healthcare professionals to report incidents. Addressing the behaviors of health workers in reporting incidents is essential to create a lesson learned from mistakes, provide valuable information to enhance a safer environment, and prevent future errors [23]. 

The Cancer Center at King Fahad Medical City is one of the main Ministry of Health tertiary cancer care facilities. It provides services to referred adult and pediatric patients from all over the Kingdom of Saudi Arabia through a referral system. The center encompasses departments of Medical Oncology, Radiation, Hematology, and Palliative care; at present, there are 94 beds for inpatients and an average of 1500 outpatient visits monthly. The patients with various hematological disorders and solid tumor malignancies were admitted or visited the clinic for diagnostic workup treatment, including chemotherapy and management of complications. Thus, this study aimed to investigate oncology staff's attitudes, perceived barriers, and strategy toward reporting incidents and errors in the oncology setting.

## Materials and methods

Study design and setting

A descriptive, cross-sectional study was conducted among healthcare professionals at Comprehensive Cancer Center in a tertiary care hospital in Riyadh, Saudi Arabia in 2019. All healthcare professionals dealing with cancer care in the center, including physicians and other allied health professional teams working at the time of the survey, were included in the study. Staff members on leave, unwilling to participate, and not working in cancer care were excluded from the study.

Procedure and ethical considerations

Data was collected using a structured online questionnaire. An email was sent to all participants explaining the study's aim, nature, and benefits. As part of taking the consent form, whenever the target participants agree on the study's conditions, they voluntarily participate and may open the web link to direct the online questionnaire. The participants were asked to complete the self-assessment survey anonymously. The confidentiality and anonymity of the participants were maintained throughout the study process. The study was approved by the ethics committee at King Fahad Medical City (approval 17-106).

Study instrument 

The questionnaire was adapted based on the review of previous related studies [24-26] and reviewed by multiple expert panels, including oncologist quality and biostatistics experts. A pilot study was conducted with 25 participants to measure the validity and clarity of the study tool throughout the test and retest analysis. The assessments of the reliability and internal consistency of the questionnaire were performed using Cronbach's alpha test, and it was estimated at 0.80.

The survey instrument is composed of four sections: (1) sociodemographic information, (2) participants' attitudes towards incidence reporting, (3) perceived hindrances, and (4) strategies for improving reporting incidence. 

The first part includes a set of questions about the participant's social demographic profile, such as gender, age, occupation type, and years of experience. In contrast, the second part was the survey attitude components, comprising 10 items, and the participants were asked to answer the questions, which were measured using the five-point Likert scale ranging from 1 to 5, 1: strongly disagree, 2: disagree, 3: neutral, 4: agree, and 5: strongly agree. The third part of the instrument included 15 survey questions about perceived hindrances to incidence reporting based on a three-point Likert scale (agree, neutral, and disagree). Additionally, the fourth part asked the respondents about the strategies to improve reporting errors.

Sample size and statistical analysis

Cochran's method was used to estimate the sample size with parameters of 95% confidence interval, 50% prevalence, and a population size of 300. It was calculated that 169 participants were needed for this study. Descriptive analysis using frequency and percentage distribution presented in frequency and percentage. Meanwhile, the analysis of variance (ANOVA)/Chi-square test was used to determine whether there is a significant association between responses to occupation and work experience. The value of P < 0.05 was considered statistically significant. SPSS version 25.0 software (IBM Corp., Armonk, NY, USA) was used in analyzing the data.

## Results

Descriptive statistics

A total number of 211 out of 250 eligible respondents participated in the study and have fully completed the questionnaire, providing an overall response rate of 84%. Most of the respondents were female (155, 73.5%), and almost half (107, 50.7%) were between the ages of 31 and 40. Nurses dominated the study population 139 (69.5%), followed by physicians 35 (16.5%), and other allied health care workers 37 (13.3%). Most respondents (125, 59.3%) had three to 10 years of work experience in cancer care (Table 1).

**Table 1:** Participants' demographic characteristics

Characteristics	n (%)
Gender	
Male	56 (26.5%)
Female	155 (73.5%)
Age	
21–30	64 (30.3%)
31–40	107 (50.7%)
41–50	36 (17.1%)
51–60	4 (1.9%)
Occupation	
Nurses	139 (65.9%)
Physicians	35 (16.6%)
Allied Health professionals	37 (17.5%)
Experience	
≤ 2 years	52 (24.6%)
3–6 years	62 (29.4%)
7–10 years	63 (29.9%)
≥ 11 years	34 (16.1%)

Attitude of healthcare professionals toward incident reporting

Most respondents responded negatively about hiding or denying a reporting error (178, 84.4%), while 138 (65.4%) felt a need to reveal errors. More than half of the responders (53%) said that they were blamed by their colleagues when an error was made, while 92 (43.6%) never had this blame. Furthermore, 112 (53.1%) participants felt that revealing an error can humiliate a colleague. Moreover, 93 (44%) respondents have also reported that healthcare practitioners should not make errors (Table 2).

**Table 2:** Attitude toward incident reporting

Statement	Strongly disagree	Disagree	Neutral	Agree	Strongly Agree
1. I would protect my self-interest ahead of the interests of the patient if I could, e.g., by hiding or denying an error.	109 (51.7%)	69 (32.7%)	14 (6.6%)	15 (7.1%)	4 (1.9%)
2. It would affect my identity as a staff to admit to an error.	74 (35.1%)	81 (38.4%)	35 (16.6%)	17 (8.1%)	4 (1.9%)
3. Others don’t need to know about the errors I have made.	68 (32.2%)	70 (33.2%)	44(20.9%)	21 (10%)	8 (3.8%)
4. Disclosing an error, if you don’t have to, is an optional act of heroism.	61 (28.9%)	77 (36.5%)	54(25.6%)	15 (7.1%)	4 (1.9%)
5. If I admit an error, I will feel like a failure.	63 (29.9%)	81 (38.4%)	41(19.4%)	21 (10%)	5 (2.4%)
6. It would affect my self-esteem to admit an error.	60 (28.4%)	82 (38.9%)	41(19.4%)	19 (9%)	9 (4.3%)
7. Healthcare practitioner who makes an error is humiliated by their colleagues.	43 (20.4%)	69 (32.7%)	57(27.0%)	30 (14.2%)	12 (5.7%)
8. Healthcare practitioners have a culture of silence where errors are not talked about.	32 (15.2%)	62 (29.4%)	74(35.1%)	31 (14.7%)	12 (5.7%)
9. Healthcare practitioner who makes an error is blamed by their colleagues.	31 (14.7%)	61 (28.9%)	66(31.3%)	37 (17.5%)	16 (7.6%)
10. Healthcare practitioners should not make errors.	37 (17.5%)	56 (26.5%)	56 (26.5%)	49 (23.2%)	13 (6.2%)

On the other hand, Table 3 describes the attitudes of healthcare professionals by occupation; however, there were no significant differences between those groups.

**Table 3:** The attitude of healthcare professionals by occupation

Statement		Nurse		Physician		Allied Health			
		n	%	n	%	n	%	p-value	
1. I would protect my self-interest ahead of the interests of the patient if I could, e.g., by hiding or denying an error.	Disagree	117	84.2	29	82.9	32	86.5	0.96	
	Neutral	10	7.2	2	5.7	2	5.4		
	Agree	12	8.6	4	11.4	3	8.1		
2. It would affect my identity as a staff to admit to an error.	Disagree	99	71.2	25	71.4	31	83.8	0.51	
	Neutral	24	17.3	6	17.1	5	13.5		
	Agree	16	11.5	4	11.4	1	2.7		
3. Others don’t need to know about the errors I have made.	Disagree	94	67.6	20	57.1	24	64.9	0.75	
	Neutral	27	19.4	10	28.6	7	18.9		
	Agree	18	12.9	5	14.3	6	16.2		
4. Disclosing an error, if you don’t have to, is an optional act of heroism.	Disagree	98	70.5	18	51.4	22	59.5	0.14	
	Neutral	28	20.1	14	40.0	12	32.4		
	Agree	13	9.4	3	8.6	3	8.1		
5. If I admit an error, I will feel like a failure.	Disagree	92	66.2	27	77.1	25	67.6	0.72	
	Neutral	28	20.1	6	17.1	7	18.9		
	Agree	19	13.7	2	5.7	5	13.5		
6. It would affect my self-esteem to admit an error.	Disagree	86	61.9	28	80.0	28	75.7	0.06	
	Neutral	29	20.9	7	20.0	5	13.5		
	Agree	24	17.3	0	0.0	4	10.8		
7. Healthcare practitioner who makes an error is humiliated by their colleagues.	Disagree	73	52.5	16	45.7	23	62.2	0.17	
	Neutral	35	25.2	15	42.9	7	18.9		
	Agree	31	22.3	4	11.4	7	18.9		
8. Healthcare practitioners have a culture of silence where errors are not talked about.	Disagree	67	48.2	10	28.6	17	45.9	0.22	
	Neutral	48	34.5	15	42.9	11	29.7		
	Agree	24	17.3	10	28.6	9	24.3		
9. Healthcare practitioner who makes an error is blamed by their colleagues.	Disagree	65	46.8	15	42.9	12	32.4	0.61	
	Neutral	40	28.8	11	31.4	15	40.5		
	Agree	34	24.5	9	25.7	10	27.0		
10. Healthcare practitioners should not make errors.	Disagree	57	41.0	17	48.6	19	51.4	0.13	
	Neutral	33	23.7	11	31.4	12	32.4		
	Agree	49	35.3	7	20.0	6	16.2		

Perceived hindrances to reporting incidences

Based on the results, 128 (60.7%) mentioned that not wanting to get into trouble is the leading barrier to reporting incidence, followed by the worried about legal actions (125, 59.2%). Moreover, respondents have also declared their worries about disciplinary action (49.3%), which hinders them from reporting the event or errors. Moreover, blaming unfairly for the event (48.3%), the reporting system taking too long to fill the report (36.3%), and no feedback after reporting the event (36.3%) are perceived as added barriers. On the other hand, 136 (64.5%) of all respondents disagree with the statement that they do not know who is responsible for making a report. Respondents' agreement with the perceived barrier's statements is shown in Table 4.

**Table 4:** Perceived hindrances to incident reporting

Statement	Disagree		Neutral		Agree	
	n	%	n	%	n	%
1. I am worried about legal action.	39	18.5	47	22.3	125	59.2
2. I don’t want to get into trouble.	39	18.5	44	20.9	128	60.7
3. My colleagues may be unsupportive.	69	32.7	58	27.5	84	39.8
4. I am worried about disciplinary action.	52	24.6	55	26.1	104	49.3
5. I may be blamed unfairly for the event.	56	26.5	53	25.1	102	48.3
6. I do not want the case discussed in meetings.	84	39.8	55	26.1	72	34.1
7. Adverse event reporting makes little contribution to the quality of care.	88	41.7	59	28	64	30.3
8. I don’t know whose responsibility it is to make a report.	136	64.5	37	17.5	38	18
9. Even if I don’t give my details, I’m worried they’ll track me down.	102	48.3	49	23.2	60	28.4
10. The electronic incident reporting system takes too long to fill in, and I just don’t have time.	84	39.8	56	26.5	71	33.6
11. When I am busy at work, I forget to make a report.	86	40.8	60	28.4	65	30.8
12. I don’t feel confident that the information I provide will be kept confidential.	90	42.7	60	28.4	61	28.9
13. I never get any feedback after I report an adverse event.	71	33.6	69	32.7	71	33.6
14. I wonder about who else will have access to the information I disclose.	66	31.3	75	35.5	70	33.2
15. As long as the healthcare practitioners involved learns from the incidents, it is unnecessary to discuss them further.	117	55.5	48	22.7	46	21.8

After stratifying by occupation, a statistically significant difference was found between physicians, nurses, and allied health in terms of barrier statements, including being blamed unfairly for the event (p-value 0.05), belittled as an adverse event (p-value 0.05), forgetting to make a report because the staff is busy at work (p-value 0.03), and confidentiality issues (p-value 0.03) as shown in Table 5.

**Table 5:** Perceived hindrances to incident reporting per occupation

Statements		Nurse		Physicians		Allied Health		
		n	%	n	%	n	%	p-value
1. I am worried about legal action.	Disagree	22	15.8	6	17.1	11	29.7	0.129
	Neutral	28	20.1	12	34.3	7	18.9	
	Agree	89	64.0	17	48.6	19	51.4	
2. I don’t want to get into trouble.	Disagree	25	18.0	7	20.0	7	18.9	0.925
	Neutral	27	19.4	8	22.9	9	24.3	
	Agree	87	62.6	20	57.1	21	56.8	
3. My colleagues may be unsupportive.	Disagree	40	28.8	12	34.3	17	45.9	0.158
	Neutral	37	26.6	13	37.1	8	21.6	
	Agree	62	44.6	10	28.6	12	32.4	
4. I am worried about disciplinary action.	Disagree	34	24.5	12	34.3	6	16.2	0.038
	Neutral	29	20.9	10	28.6	16	43.2	
	Agree	76	54.7	13	37.1	15	40.5	
5. I may be blamed unfairly for the event.	Disagree	34	24.5	12	34.3	10	27.0	0.051
	Neutral	37	26.6	12	34.3	4	10.8	
	Agree	68	48.9	11	31.4	23	62.2	
6. I do not want the case discussed in meetings.	Disagree	49	35.3	16	45.7	19	51.4	0.301
	Neutral	37	26.6	8	22.9	10	27.0	
	Agree	53	38.1	11	31.4	8	21.6	
7. Adverse event reporting makes little contribution to the quality of care.	Disagree	53	38.1	22	62.9	13	35.1	0.057
	Neutral	38	27.3	7	20.0	14	37.8	
	Agree	48	34.5	6	17.1	10	27.0	
8. I don’t know whose responsibility it is to make a report.	Disagree	98	70.5	19	54.3	19	51.4	0.11
	Neutral	19	13.7	8	22.9	10	27.0	
	Agree	22	15.8	8	22.9	8	21.6	
9. Even if I don’t give my details, I’m worried they’ll track me down.	Disagree	61	43.9	17	48.6	24	64.9	0.157
	Neutral	32	23.0	10	28.6	7	18.9	
	Agree	46	33.1	8	22.9	6	16.2	
10. The electronic incident reporting system takes too long to fill in, and I just don’t have time.	Disagree	61	43.9	8	22.9	15	40.5	0.233
	Neutral	35	25.2	12	34.3	9	24.3	
	Agree	43	30.9	15	42.9	13	35.1	
11. When I am busy at work, I forget to make a report.	Disagree	55	39.6	9	25.7	22	59.5	0.035
	Neutral	41	29.5	10	28.6	9	24.3	
	Agree	43	30.9	16	45.7	6	16.2	
12. I don’t feel confident that the information I provide will be kept confidential.	Disagree	50	36.0	19	54.3	21	56.8	0.033
	Neutral	40	28.8	11	31.4	9	24.3	
	Agree	49	35.3	5	14.3	7	18.9	
13. I never get any feedback after I report an adverse event.	Disagree	53	38.1	10	28.6	8	21.6	0.324
	Neutral	41	29.5	12	34.3	16	43.2	
	Agree	45	32.4	13	37.1	13	35.1	
14. I wonder about who else will have access to the information I disclose.	Disagree	49	35.3	11	31.4	6	16.2	0.257
	Neutral	46	33.1	13	37.1	16	43.2	
	Agree	44	31.7	11	31.4	15	40.5	
15. As long as the healthcare practitioners involved learns from the incidents, it is unnecessary to discuss them further.	Disagree	83	59.7	17	48.6	17	45.9	0.111
	Neutral	25	18.0	13	37.1	10	27.0	
	Agree	31	22.3	5	14.3	10	27.0	

Strategies to improve incident reporting compliance ranked by occupation

Table 6 shows the responses to the statements for the best strategies toward improving incident reporting compliance by participant occupation. Nurses said that the precise guidelines for error reporting (114) are the first strategies to improve incident reporting, followed by the ability to report anonymously (112), then individualized feedback after submitting a report (112). Meanwhile, doctors said that the purpose of reporting (32), which is the first strategy of improving incidence, reporting following giving feedback about medical error reports (31), and seniors encouraging error reporting (31). In addition to this, the top strategies chosen by allied healthcare were education about the purpose of reporting (31), training in electronic incident reporting systems (31), and clear guidelines for error reporting (30).

**Table 6:** Strategies to improve incident reporting compliance

Statements	Nurse		Physician		Allied Health	
	Frequency	Rank	Frequency	Rank	Frequency	Rank
1. Give generalized feedback about medical error reports.	108	5	31	2	27	4
2. Role models, e.g., senior colleagues, and departmental directors who openly encourage reporting.	111	3	31	2	26	5
3. Acquire legal protection of information provided for the purpose of court investigation.	105	8	29	4	28	3
4. Ability to report anonymously.	112	2	24	8	18	9
5. Clear guidelines about what adverse events and errors to report.	114	1	30	3	30	2
6. Information on how confidentiality will be maintained if you supply your name.	109	4	28	5	23	8
7. Individualized feedback to you about the reports you submit.	112	2	27	6	25	6
8. More support from colleagues.	106	7	26	7	24	7
9. Less blame is attached to those who report errors.	97	9	27	6	23	8
10. Education about the purpose of reporting.	111	3	32	1	31	1
11. Further training on how to use the electronic incident reporting system.	107	6	26	7	31	1

## Discussion

This study offered a unique opportunity to examine healthcare professionals' attitudes and perceived hindrances. In addition, it addresses strategies to enhance the incident reporting culture for healthcare professionals at an oncology center in Saudi Arabia. Moreover, the significance of this study is to provide the necessary information to empower decision-makers seeking to improve the incident reporting culture within the field of oncology.

Our study revealed that most nurses (67.6%), physicians (57.1%), and allied healthcare professionals (64.9%) agreed to report errors. A similar study conducted in six South Australian hospitals revealed similar positive attitudes from physicians and nurses [3]; however, nurses reported errors more often than physicians because of a culture difference [17]. Moreover, around 44% of respondents reported not feeling blamed by colleagues after an error. A similar study sought to determine attitudes toward incident reporting and found that 42.4% of health practitioners felt a non-punitive culture of reporting in their workplace [6]. 

Medical errors are inevitable, but expecting the unavoidable does not mean succumbing patients to preventable errors and exposing institutional reputations to greater risk. A patient should never be harmed by the care that the patient was told to receive. Enhancing clinical coordination between multidisciplinary teams can help uplift the patient safety culture. Moreover, full engagement by interdisciplinary teams in patient admission, handover, and discharge connects the planning process with a review of the risks and benefits of certain medical decisions. Through integrating multidisciplinary teams, errors can be detected, and the mindset can shift toward a willingness to report incidents. 

Healthcare providers consider patient safety a top priority and responsibility. Organizing a process for active incident reporting is the best way to reconfigure our culture of safety. A study showed that physicians are the least likely professionals to report safety issues unless an incident violates established policy or protocol. Moreover, most healthcare staff report incidents to colleagues rather than through the organization's incident reporting system [8,10]. This behavior creates a significant risk for patients. 

Several studies have explored barriers to incident reporting in healthcare settings [27]. These barriers are eight of 10 different between organizations [10]. According to health professionals' perception, healthcare workers tend not to report incidents during their practice or duty because they feel such actions can impact them negatively. According to our findings, 60.7% of respondents did not report it because it might cause personal trouble. This finding is like previous research revealing that organizational culture can negatively influence rates of incident reporting [20]. Other perceived barriers have been possible legal ramifications or disciplinary actions. In our study, 59.2% of respondents were worried about legal actions, and nearly half were concerned about disciplinary action because of making a report. Like previously published research, "the presence of a punitive response to an error" at an organization can affect whether an incident is reported [21]. Furthermore, there were significant differences between nurses, physicians, and allied healthcare professionals regarding barriers related to being blamed unfairly for the event (p-value 0.05), belittling (p-value 0.05), forgetting to make a report when busy (p-value 0.03), and confidentiality issues (p-value 0.03). 

Participants suggested several strategies to improve compliance with incident reporting. Based on our findings, physicians and allied healthcare professionals strongly preferred education to report errors, while nurses preferred having clear guidelines. This indicates that nurses prefer a more structured approach than the other two groups, even while reporting incidents. Nurses are trained to follow strict medical guidelines and protocols, evident in their reporting culture. Additionally, all respondents' groups agreed that obtaining added peer support is a good strategy, proving that teamwork is vital in any practice. Similar studies show that creating the right environment is part of the success of an organization. Specifically, having a common goal and supportive peers makes a synergetic climate that can produce a team to achieve a unified vision [28], and this is important to address safety concerns like reporting incidents that occur at the workplace. 

The study results revealed significant strategies to enhance reporting events and errors, such as supplying clear guidelines about adverse events and errors to report, the ability to report anonymously, providing feedback about submitted reports, encouraging reporting, and preventing palming to those who report errors, providing education and training about the purpose of reporting, and on how to use an electronic incident reporting system.

The study had a few limitations, like this study was conducted at one center. Another limitation is that the number of reported errors was not evaluated to be compared with perceived attitudes and barriers to reporting errors. Nevertheless, this study provides important insights into healthcare providers' attitudes, hindrances, and strategies for reporting incidents and errors in the cancer setting. We encourage further detailed studies to be carried out across healthcare cancer institutes nationwide to address this crucial issue of reporting errors. Furthermore, it emphasizes the need to implement education and training programs for healthcare professionals to report errors.

## Conclusions

Adverse events and errors are preventable, and healthcare professionals should be proactive in reporting events and errors to enhance patient safety in healthcare organizations. Therefore, healthcare organizations must support and implement patient safety programs like having anonymous error reporting systems and building non-punitive atmospheres to ensure patient safety concerns and feedback are reported. Furthermore, as addressed in our study, standardized strategies to overcome barriers will help decision-makers make the necessary investment in creating the most impactful patient safety program.
